# Soluble RANKL contributes to osteoclast formation in adult mice but not ovariectomy-induced bone loss

**DOI:** 10.1038/s41467-018-05244-y

**Published:** 2018-07-25

**Authors:** Jinhu Xiong, Keisha Cawley, Marilina Piemontese, Yuko Fujiwara, Haibo Zhao, Joseph J. Goellner, Charles A. O’Brien

**Affiliations:** 1 Center for Musculoskeletal Disease Research, University of Arkansas for Medical Sciences, Little Rock, 72205 AR USA; 2 Department of Orthopaedic Surgery, University of Arkansas for Medical Sciences, Little Rock, 72205 AR USA; 3 Division of Endocrinology, University of Arkansas for Medical Sciences, Little Rock, 72205 AR USA; 4 Central Arkansas Veterans Healthcare System, Little Rock, 72205 AR USA

## Abstract

Receptor activator of NFkB ligand (RANKL) is a TNF-family cytokine required for osteoclast formation, as well as immune cell and mammary gland development. It is produced as a membrane-bound protein that can be shed to form a soluble protein. We created mice harboring a sheddase-resistant form of RANKL, in which soluble RANKL is undetectable in the circulation. Lack of soluble RANKL does not affect bone mass or structure in growing mice but reduces osteoclast number and increases cancellous bone mass in adult mice. Nonetheless, the bone loss caused by estrogen deficiency is unaffected by the lack of soluble RANKL. Lymphocyte number, lymph node development, and mammary gland development are also unaffected by the absence of soluble RANKL. These results demonstrate that the membrane-bound form of RANKL is sufficient for most functions of this protein but that the soluble form does contribute to physiological bone remodeling in adult mice.

## Abstract (web summary)

RANKL is a cytokine produced as a membrane-bound and a secreted protein. Here, using mice lacking soluble RANKL, the authors show that the secreted protein is important for osteoclast function, but not for mammary gland and lymphocyte development.

## Introduction

Physiological and pathological bone resorption by osteoclasts require RANKL, a protein encoded by the *Tnfsf11* gene^1,2^. RANKL produced by osteoblast-lineage cells binds to its receptor RANK on the surface of myeloid cells stimulating their differentiation into osteoclasts^3^. In addition to its role in bone resorption, RANKL contributes to lymphocyte production and is required for lymph node development, the final stages of mammary gland development, and microfold cell differentiation in the intestine^1,4,5^. RANKL is initially produced as a type II transmembrane protein that can be cleaved by proteases to yield a soluble form (sRANKL)^6^. The levels of circulating sRANKL are often elevated in conditions such as sex steroid deficiency, periodontitis, cancer, and inflammatory joint disease^7–11^. However, it is unclear whether sRANKL is functionally involved in osteoclast formation in these or normal physiological conditions. To address this question, we created mice lacking sRANKL and examined the impact of this change on physiological and pathological bone resorption, as well as other functions of RANKL. We find that while most functions of RANKL are unaffected, osteoclast number is reduced and bone mass is increased in adult mice lacking sRANKL.

## Results

### Creation of mice lacking sRANKL

**Fig. 1:**
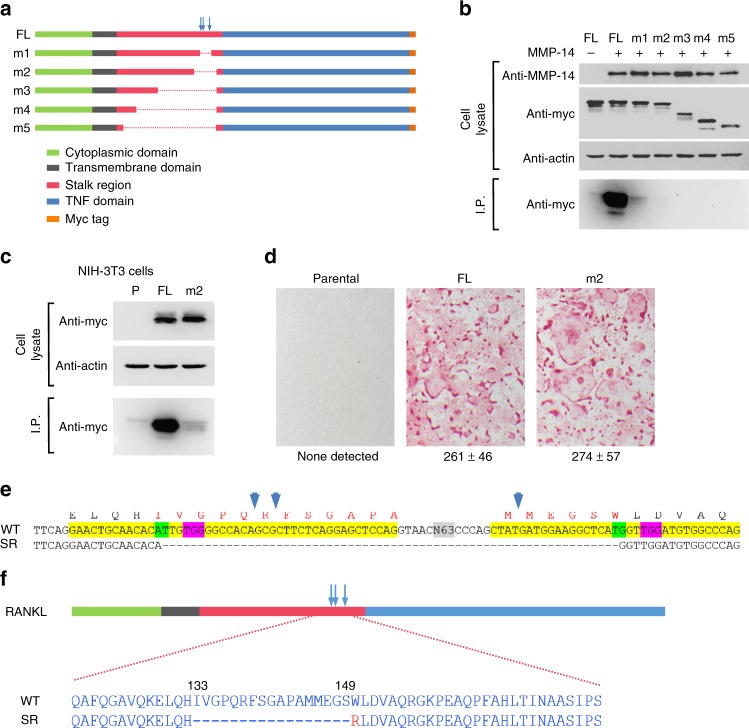
Development of a sheddase-resistant RANKL. **a** Diagram of full-length (FL) and mutant RANKL constructs. Known cleavage sites are indicated by vertical arrows. **b** Sheddase assay in transiently transfected 293T cells. This experiment was replicated at least once. **c** Sheddase assay in stably transduced NIH-3T3 cells. P parental NIH-3T3 cells. This experiment was replicated at least once. **d** Osteoclast formation in co-cultures of bone marrow macrophages with the indicated NIH-3T3 cells shown in “**c**”. Osteoclasts are stained red. Quantification of multinucleated osteoclasts from triplicate wells shown below, mean ± s.d. This experiment was replicated at least once. **e** Partial DNA sequence of murine *Tnfsf11* gene showing CRISPR/Cas9 gene-editing strategy. Exon 3 and a portion of exon 4 are highlighted in yellow. The sequence of wild-type (WT) and sheddase-resistant (SR) mutants are shown. N63 indicates 63 bp of intron 3 not shown. The PAM sequences for each of the two single-guide RNA (sgRNA) targets are highlighted in pink and their respective cut sites highlighted in green. Amino acids encoded by the exons are shown above the DNA sequence with the locations of known proteolytic cleavage sites indicated by the vertical blue arrows. The SR mutant was created by non-homologous end-joining of the double-stranded DNA cuts directed by the sgRNAs. **f** Diagram of a portion of the WT and SR RANKL proteins highlighting the amino acid sequences in the cleavage region. The red R indicates the position of an arginine resulting from deletion of the sequence shown in “**e**”

To generate a form of RANKL resistant to proteolytic cleavage, we sought to create a deletion mutant that exhibited reduced levels of shedding but retained the ability to support osteoclast formation when expressed on the surface of stromal cells. We created a series of deletion constructs lacking increasing amounts of the stalk region, which contains all known cleavage sites^12–14^ (Fig. 1a). In addition, we attached a myc epitope tag to the carboxy-terminus of the protein to allow detection of the shed RANKL by immunoprecipitation and subsequent immunoblotting with an anti-myc antibody. We then tested the ability of these mutants to resist the action of MMP14, a protease that exhibits potent RANKL sheddase activity, in transfected 293T cells^14^. Deletion of the region from Arg^138^ to Met^146^ (construct m1) reduced but did not eliminate shedding whereas deletion of the region from Ile^133^ to Leu^151^ (construct m2) appeared to abolish shedding (Fig. 1b).

We selected the m2 construct for further characterization since it was the longest of the relatively more sheddase-resistant (SR) mutants and thus more likely to retain osteoclast support activity. Full-length RANKL and m2 were stably transduced into the NIH-3T3 cell line, which exhibited potent sheddase activity towards the full-length RANKL, even in the absence of co-transfected proteases (Fig. 1c). However, m2 was resistant to these endogenous sheddases (Fig. 1c). Since NIH-3T3 cells do not express *Tnfsf11*, we used the transduced cells to confirm the ability of the SR mutant to support osteoclast formation in co-cultures of the transduced NIH-3T3 cells with osteoclast progenitors (Fig. 1d). Based on these results, we used the CRISPR/Cas9 system to modify the endogenous murine *Tnfsf11* gene to produce a similar SR protein (Fig. 1e). The protein encoded by the modified allele, designated *Tnfsf11*^SR^, lacks amino acids Ile^133^ to Ser^149^ and contains Arg at the former Trp^150^ position (Fig. 1f).

### Physiological role of sRANKL

**Fig. 2:**
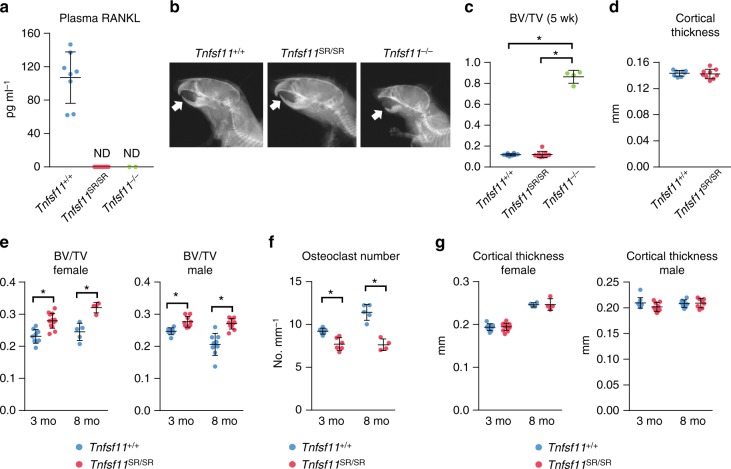
Soluble RANKL contributes to osteoclast formation in adult mice. **a** sRANKL levels in the blood plasma of 5-week-old Tnfsf11^+/+^ (*n* = 8), Tnfsf11^SR/SR^ (*n* = 9), and Tnfsr11^−/−^ (*n* = 2) mice. ND not detectable. **b** X-ray images from 5-week-old Tnfsf11^+/+^, Tnfsf11^SR/SR^, and Tnfsr11^−/−^ mice. Arrowheads indicate position of lower incisors. **c** Cancellous bone volume of L4 vertebra from 5-week-old Tnfsf11^+/+^ (*n* = 8), Tnfsf11^SR/SR^ (*n* = 9), and Tnfsr11^−/−^ (*n* = 4) mice. *P < 0.05 versus Tnfsf11^+/+^ or Tnfsf11^SR/SR^ mice using one-way ANOVA. **d** Cortical thickness of femurs from 5-week-old Tnfsf11^+/+^ (*n* = 8) and Tnfsf11^SR/SR^ (*n* = 9) mice. **e** Cancellous bone volume of L4 vertebra from 3-month-old female Tnfsf11^+/+^ (*n* = 9) and Tnfsf11^SR/SR^ (*n* = 13) and male Tnfsf11^+/+^ (*n* = 8) and Tnfsf11^SR/SR^ (*n* = 9) mice and 8-month-old female Tnfsf11^+/+^ (*n* = 5) and Tnfsf11^SR/SR^ (*n* = 4) and male Tnfsf11^+/+^ (*n* = 9) and Tnfsf11^SR/SR^ (*n* = 10) mice. *P < 0.05 versus Tnfsf11^+/+^ mice using Student’s *t* test. **f** Osteoclast number per mm bone surface measured in the cancellous bone of L1–L3 vertebra of 3-month-old Tnfsf11^+/+^ (*n* = 6) and Tnfsf11^SR/SR^ (*n* = 6), and 8-month-old Tnfsf11^+/+^ (*n* = 5) and Tnfsf11^SR/SR^ (*n* = 4), female littermates. *P < 0.05 using Student’s *t* test. **g** Cortical thickness of femurs from 3-month-old female Tnfsf11^+/+^ (*n* = 9) and Tnfsf11^SR/SR^ (*n* = 13) and male Tnfsf11^+/+^ (*n* = 9) and Tnfsf11^SR/SR^ (*n* = 9) mice and 8-month-old female Tnfsf11^+/+^ (*n* = 5) and Tnfsf11^SR/SR^ (*n* = 4) and male Tnfsf11^+/+^ (*n* = 9) and Tnfsf11^SR/SR^ (*n* = 10) mice. All analyses of 5-week-old mice included both sexes. All values are means ± s.d.

Similar to *Tnfsf11*^*−/−*^ mice, sRANKL was undetectable in the blood plasma of *Tnfsf11*^SR/SR^ mice by ELISA, demonstrating that the SR mutation potently inhibits RANKL shedding in vivo (Fig. 2a). We confirmed the results of the ELISA using affinity isolation of sRANKL with a RANK-Fc fusion protein followed by immunoblotting (Supplementary Fig. 1). The process of tooth eruption, which requires osteoclasts, was completely inhibited in *Tnfsf11*^−/−^ mice but unaffected in *Tnfsf11*^SR/SR^ mice (Fig. 2b). Similarly, *Tnfsf11*^SR/SR^ mice displayed normal levels of T- and B-lymphocytes, as well as development of lymph nodes (Supplementary Fig. 2). At 5 weeks of age, *Tnfsf11*^SR/SR^ mice had bone mass that was indistinguishable from wild-type littermates (Fig. 2c, d). However, at 3 and 8 months of age, *Tnfsf11*^SR/SR^ mice exhibited elevated cancellous bone volume in both sexes and this was associated with reduced osteoclast number in cancellous bone (Fig. 2e, f). On the other hand, cortical thickness was unaffected (Fig. 2g). These results demonstrate that the membrane-bound form of RANKL is sufficient for osteoclast formation in the developing and growing skeleton but that as the skeleton matures, sRANKL makes an essential contribution to osteoclast formation in cancellous bone.

### Role of sRANKL in estrogen deficiency

**Fig. 3:**
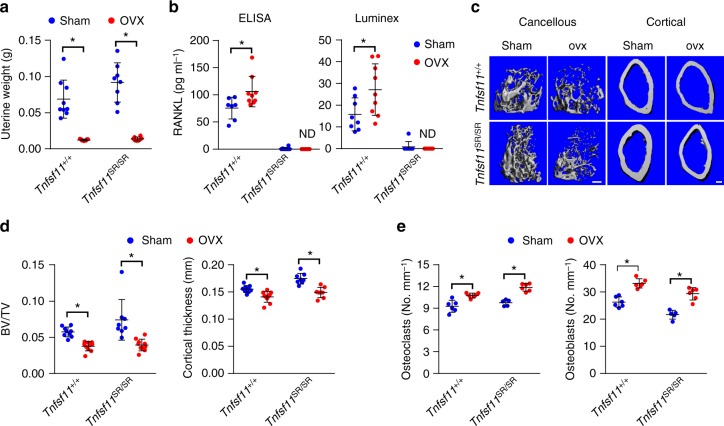
Soluble RANKL is not required for the bone loss caused by estrogen deficiency. **a** Uterine weight from Tnfsf11^+/+^ (sham *n* = 9, ovx *n* = 10) and Tnfsf11^SR/SR^ (sham *n* = 8, ovx *n* = 8) mice. **b** Soluble RANKL in serum from Tnfsf11^+/+^ (sham *n* = 7, ovx *n* = 9) and Tnfsf11^SR/SR^ (sham *n* = 8, ovx *n* = 7) mice measured by ELISA (left) or from Tnfsf11^+/+^ (sham *n* = 8, ovx *n* = 9) and Tnfsf11^SR/SR^ (sham *n* = 8, ovx *n* = 8) mice measured by Luminex assay (right). **c** µCT images of femoral cancellous and cortical bone. Scale bar = 200 μm. **d** Cancellous bone volume (left) of femurs from Tnfsf11^+/+^ (sham *n* = 9, ovx *n* = 10) and Tnfsf11^SR/SR^ (sham *n* = 8, ovx *n* = 10) mice and cortical thickness (right) of femurs from Tnfsf11^+/+^ (sham *n* = 9, ovx *n* = 10) and Tnfsf11^SR/SR^ (sham *n* = 8, ovx *n* = 8) mice. **e** Osteoclast number (left) and osteoblast number (right) per mm bone surface measured in vertebral cancellous bone from Tnfsf11^+/+^ (sham *n* = 6, ovx *n* = 6) and Tnfsf11^SR/SR^ (sham *n* = 5, ovx *n* = 6) mice. All values in Fig. 3 are from female mice sham-operated or ovariectomized at 3 months of age and analyzed 6 weeks later. All values are means ± s.d. *P < 0.05 versus sham-operated controls of the same genotype by Student’s *t* test

Previous studies have demonstrated that sex steroid deficiency in rodents increases the production of sRANKL^15,16^, suggesting that sRANKL may participate in the increased osteoclast formation that occurs in this condition. To investigate this possibility, we performed sham operations or ovariectomies in 3-month-old *Tnfsf11*^SR/SR^ and wild-type mice. It is important to note that the wild-type mice in this experiment were purchased from a vendor. Therefore, we compared the response of the two genotypes to ovariectomy but did not compare their basal skeletal phenotype. After 6 weeks, uterine weight was reduced in both ovariectomized groups, confirming estrogen deficiency (Fig. 3a). Soluble RANKL in the circulation was higher in ovariectomized wild-type mice as measured by two different immunoassays (Fig. 3b). In contrast, sRANKL remained extremely low or undetectable in *Tnfsf11*^SR/SR^ mice (Fig. 3b). Osteoprotegerin (OPG) levels were unchanged by ovariectomy in either genotype (Supplementary Fig. 3). Ovariectomy reduced femoral cancellous bone volume and cortical thickness in wild-type mice and similar decreases occurred in *Tnfsf11*^SR/SR^ mice (Fig. 3c, d). Consistent with the changes in bone mass, estrogen deficiency increased cancellous bone remodeling similarly in both genotypes, as measured by osteoclast and osteoblast number in vertebral cancellous bone (Fig. 3e). These results demonstrate that although sRANKL production increases during estrogen deficiency, it is not required for the increase in osteoclasts or bone loss associated with this condition.

## Discussion

The successful eruption of teeth and normal bone mass of young *Tnfsf11*^SR/SR^ mice suggests that sRANKL is not required for the majority of the osteoclast formation that occurs in growing mice. Soluble RANKL is also not required for lymph node development or the development of T and B lymphocytes. We also noted that female *Tnfsf11*^SR/SR^ mice were able to successfully nurse offspring (not shown), consistent with normal mammary gland development. In addition, Takayanagi and colleagues recently demonstrated that sRANKL is not required for microfold cell development in the intestine using an allele similar to the one described here^17^. Thus, it appears that the membrane-bound form of RANKL is sufficient for osteoclastogenesis during normal skeletal development and growth as well as many of its other physiological functions.

It has been suggested that shedding of RANKL may be a mechanism that limits osteoclast formation^14^. This idea is based on the high level of osteoclast formation in mice lacking MMP14, a matrix metalloproteinase with a demonstrated ability to promote RANKL shedding^14,18^. Our finding that osteoclast formation was reduced in mice lacking sRANKL argues against this idea and suggests that the increased bone resorption in MMP14-null mice results from changes in MMP14 targets other than RANKL.

As with RANKL, many other members of the TNF family of cytokines are shed to produce soluble forms. For the most part, the functional role of membrane-bound versus soluble forms has been examined in vitro or by transgenic over-expression of mutants that are either soluble or restricted to the membrane^19–22^. However, the only other TNF family cytokine gene modified to produce only the membrane-bound form is *Tnf*^20^. Similar to our *Tnfsf11* mutation, deletion of the protease target sites from the murine *Tnf* gene completely inhibited the production of soluble TNF but did not alter the function of the membrane-bound form^20^. Analysis of these mice revealed that the membrane-bound form is sufficient for many of the physiological functions of TNF but the soluble form is required for optimal inflammatory lesion development^20^. Importantly, studies using these mice confirmed some, but not all, of the functions ascribed to membrane-bound TNF based on in vitro studies^20,23^. These studies, together with the present work, highlight the importance of using endogenous gene modification to determine the functions of soluble versus membrane-bound forms of TNF cytokines.

In contrast to growing mice, we found that sRANKL does contribute to osteoclast formation and bone remodeling in adult mice. We and others have shown that osteocytes are an essential source of the RANKL that drives remodeling of cancellous bone in adult mice but are not an important source in young growing mice^3,24^. The similarity of the phenotypes of the two models suggests that osteocytes communicate with osteoclast progenitors, in part, via production of sRANKL. It is important to note, however, that the magnitude of the reduction in osteoclast number in mice lacking sRANKL was smaller than in mice lacking osteocyte RANKL, indicating that osteocytes also utilize the membrane-bound form to support osteoclast formation. Since most osteocytes do not appear to make direct contact with either blood vessels or the bone marrow^25^, how membrane-bound RANKL in osteocytes makes contact with osteoclast progenitors is unclear. This issue becomes even more pronounced when considering larger animals with true osteonal remodeling, in which the vast majority of osteocytes are buried deeply in bone^26^. One possibility is that the contribution of sRANKL is relatively greater in these larger animals compared to mice. This question, and the role of sRANKL in pathological conditions other than estrogen deficiency, will require additional study.

## Methods

### DNA constructs

Plasmids for expression of myc-tagged RANKL in 293T cells (CRL-3216, ATCC) were derived from pUNO1-mTRANCEa (InvivoGen). Two myc tag epitopes were inserted at the C-terminus of the RANKL coding sequence using a Geneblock (Integrated DNA technologies). Deletion mutants were created by inserting synthetic DNA fragments encoding the truncations between the BsrBI and BglII sites of pUNO1-mTRANCEa-myc. Plasmids for retroviral transduction of full-length and truncated RANKL into NIH-3T3 cells were prepared by moving the RANKL-encoding cDNAs from pUNO1 into the vector pST^27^. Plasmids for expression of Cas9 and sgRNAs in developing embyros were prepared by inserting oligonucleotides encoding the desired sgRNA sequence into the pX330 vector using the protocol recommended by the Zhang laboratory^28^. All constructs were verified by DNA sequencing. 293T cells were cultured in DMEM (Sigma) containing 10% FBS and transfected using TransIT-LT1 (Mirus Bio, LLC). Production of retroviral particles was performed by transient transfection of a variant of 293T cells known as Phoenix-ampho^29^ and the resulting supernatants were used to infect NIH-3T3 cells (CRL-1658, ATCC) in the presence of hexadimethrine bromide (4 µg ml^−1^)^27^. Cell lines were not tested for mycoplasma but were routinely cultured in Plasmocin (Invivogen), an anti-mycoplasma antibiotic.

### Immunoprecipitation assay

Myc-tagged RANKL proteins were immunoprecipitated from culture supernatants via the following procedure. Confluent monolayers of transiently transfected 293T cells or transduced NIH-3T3 cells in six-well plates were cultured with 2.5 ml of fresh medium for 24 h after which the conditioned medium was collected and centrifuged at 1500 × *g* for 5 min at room temperature. The supernatant was transferred to a fresh tube and incubated with 40 µl of agarose beads covalently bound to an anti-myc monoclonal antibody (9E10 AC, Santa Cruz Biotechnology). After rotating overnight at 4 °C, the beads were washed twice with PBS and proteins were eluted by boiling in 30 µl of 2× SDS sample buffer. Eluted proteins were then analyzed by immunoblotting.

### Immunoblot

Proteins were isolated from the whole cells using the RIPA buffer (Sigma-Aldrich, St. Louis, MO) supplemented with protease inhibitor cocktail (Thermo Fisher Scientific, MA) or obtained from an immunoprecipitation assay. The concentrations of protein samples from cell extracts were determined by the BCA protein assay (Thermo Fisher Scientific). Proteins were resolved in SDS-polyacrylamide gels and electroblotted onto polyvinylidene difluoride membranes. The membranes were blocked in TBS with 5% non-fat dry milk and then were incubated with primary antibodies overnight at 4 °C and an appropriate horseradish peroxidase-linked secondary antibody at room temperature for 1 h. The following antibodies were used at a 1–1000 dilution: anti-RANKL (AF462, R&D systems, Minneapolis, MN); anti-mmp14 (ab51074, Abcam, Cambridge, MA); anti-myc (9E10, Santa Cruz Biotechnology); and anti-β-actin (sc-81178, Santa Cruz Biotechnology). Uncropped blots are presented in Supplementary Figs. 1, 5, and 6.

### Osteoclast culture

Bone marrow cells were isolated from the femurs and tibias of 4-month-old male C57BL/6 mice and were cultured in a petri dish in αMEM supplemented with 15% FBS and 100 ng ml^−1^ M-CSF for 6 days to generate macrophages. Bone marrow macrophages were then co-cultured with NIH3T3 cells expressing different RANKL constructs in αMEM supplemented with 10% FBS and 10 ng ml^−1^ M-CSF for 4 days to generate osteoclasts. The culture medium was changed every day. At day 4, the cells were fixed in 10% buffered formalin and osteoclasts were visualized by TRAP staining.

### Generation of Tnfsf11 SR mice

Plasmids expressing Cas9 and sgRNAS (pX330) were injected into the pronuclei of fertilized C57BL/6 mouse eggs at a final concentration of 4 ng µl^−1^. Microinjected eggs were then implanted in the oviducts of pseudopregnant ICR mice. Founders were screened for the desired mutation using the following primers: forward 5ʹ-GCATCTGGTAGTCCTGCGTA-3ʹ; reverse 5ʹ-CAGGCTGGGTGGAAATGTAA-3ʹ; product size 214 bp for the SR allele, and 352 bp for the wild-type allele. Offspring harboring the pX330 plasmid were identified by PCR and not used for further breeding. Possible off-target mutations were identified by a PCR-based screen of the top six potential off-target cut sites identified using the MIT CRISPR design tool (http://crispr.mit.edu/) (Supplementary Tables 1–3). A single off-target mutation was identified in the founder line used for these studies and it was eliminated from the colony by selective breeding. Breeding of the founder mouse and all subsequent offspring was performed with C57BL/6J mice.

### Quantification of sRANKL and OPG

Blood was collected by retro-orbital bleeding into heparinized capillary tubes and was centrifuged at 1500 × *g* for 10 min to separate plasma from cells. Soluble RANKL and OPG in blood plasma were measured using mouse Quantikine kits (R&D Systems) according to the manual provided by the manufacturer. For some experiments, RANKL protein levels were determined using the Procarta immunoassay kit (Affymetrix, Santa Clara, CA, USA). For these studies, data were acquired using a Luminex-200 reader (Luminex, Austin, TX, USA). A five-parameter regression formula was used to calculate the sample concentrations from standard curves using Luminex xPONENT 3.1 software.

### Affinity isolation

Blood samples were collected from *Tnfsf11*^SR/SR^ and control mice by retro-orbital bleeding. The blood samples were incubated at room temperature for 1 h to clot and the serum was collected after centrifuge at 500 × *g* for 10 min. The serum samples were pooled for each genotype and 2 ml of serum was used for the following RANKL affinity isolation. Serum was incubated with Protein A-Sepharose 4B (101090, Thermo Fisher Scientific) for 5 h at 4 °C to pre-absorb immunoglobulins. RANK-Fc (683-RK-100, R&D systems) or IgG (sc-2025, Santa Cruz) was then coupled to Protein A-Sepharose 4B and incubated with the pre-absorbed serum samples at 4 °C overnight. The agarose beads were washed with PBST for four times and then were boiled for 5 min in 30 µl 2× SDS sample buffer to elute proteins. RANKL protein was then detected by immunoblot using anti-RANKL antibody (AF462, R&D systems).

### Flow cytometry

Bone marrow cells were collected by removing both ends of femurs and flushing out the cells with PBS containing 3% FBS. Single-cell suspensions of spleen cells were prepared by grinding the spleen through a cell strainer using a syringe plunger. Bone marrow and spleen cells were then washed and incubated with anti-mouse CD16/CD32 (mouse BD Fc-block, catalog number 553141, BD Biosciences, San Jose, CA) for 5 min. The cells were stained for 30 min using the following antibodies: 2 µg ml^−1^ anti-CD19-APC-Cy7 (1D3, BD Biosciences) to identify B cells and 5 µg ml^−1^ anti-CD3-FITC (145-2C11, BD Biosciences) to identify T cells. After the cells were washed to remove unbound antibodies, the samples were analyzed by a BD FACS Aria flow cytometer (BD Biosciences). An example of the gating strategy is presented in Supplementary Fig. 4. The data were then analyzed using FlowJo Software (FlowJo, LLC, Ashland, OR). The guidance of Fluorescence Minus One (FMO) controls (BD Biosciences) was followed to draw appropriate gates for the cell populations.

### Lymph node analysis

Inguinal lymph nodes were isolated from mice and were fixed in 10% Millonig’s formalin overnight. After fixation, lymph nodes were soaked in 30% sucrose for 2 days and then embedded in Cryo-Gel (Electron Microscopy Sciences, Hatfield, PA) for sectioning. Five micron frozen sections were made using a Leica CM3050S cryostat (Leica Biosystems, Buffalo Grove, IL). The sections were air dried at room temperature for 1 h and then stained with hematoxylin and eosin for imaging.

### Ovariectomy

Three-month-old female *Tnfsf11*^SR/SR^ mice or 3-month-old female wild-type C57BL/6 mice obtained from the National Institute of Aging were either sham-operated or ovariectomized. Mice were assigned to ovariectomy or sham groups based on bone mineral density (BMD) of the lumbar spine. Specifically, mice were rank-ordered by BMD and then assigned the number 1 or 2, successively. Animals with the same number were assigned to the same operation group to give similar group means^2^. After 6 weeks, mice were euthanized and the tissues were dissected for further analyses. BMD was measured prior to surgery and before euthanasia. All animal procedures were reviewed and approved by the Institutional Animal Care and Use Committee of the University of Arkansas for Medical Sciences.

### X-ray

Mice were anesthetized by injection of Nembutal (80 mg kg^−1^ body weight) intraperitoneally. Mice were then placed on top of radiographic film and exposed to X-rays for 13 s at 40 kV in an AXR 110 Minishot X-ray machine (Associated X-Ray Corporation, East Haven, CT).

### BMD determination

BMD was determined using a PIXImus densitometer (GE-Lunar Corp., Madison, WI) running software version 2.0. Three sites were measured. The total body window was defined as the whole body image minus the calvarium, mandible, and teeth. Except for the first few caudal vertebrae, the tail was not included. The spine window was a rectangle depending on animal body length, reaching from just below the skull to the base of the tail. The femoral window captured the right femur. Scan acquisition time was 4 min and analysis time was 6 min. The mice were sedated during scanning with isoflurane to keep the animals motionless for the required 4 min and to facilitate rapid post examination recovery. The animals were monitored by observation of the righting reflex, respiration, and heart rate. Using a proprietary skeletal phantom, measurements of the total body BMD performed over the past 4 years had a mean coefficient of variation of 3.1% (*n* = 285).

### MicroCT

MicroCT scanning was used to measure cortical and trabecular architecture of the fourth lumbar vertebra and femur. L4 vertebra and femurs were dissected, cleaned of soft tissues, fixed in 10% Millonig’s formalin overnight, and transferred gradually from 70 to 100% ethanol. Dehydrated bones were then loaded into a 12.3-mm-diameter scanning tube and scanned by a µCT (model µCT40, Scanco Biomedical, Bruttisellen, Switzerland) to generate three-dimensional voxel images (1024 × 1024 pixels) of bone samples. A Gaussian filter (sigma = 0.8, support = 1) was used to reduce signal noise and a threshold of 200 was applied to all scans, at medium resolution (*E* = 55 kVp, *I* = 145 µA, integration time = 200 ms). For vertebra, the whole vertebral body was scanned, and cortical bone and the primary spongiosa were excluded from the analyses manually. For femurs, 151 slices above the growth plate of the distal femur were scanned for trabecular measurement and the top 50 slices were analyzed for cortical thickness. All trabecular measurements were made by drawing contours every 10–20 slices and using voxel counting for bone volume per tissue volume and sphere-filling distance transformation indices, without presumptions about the bone shape as a rod or plate for trabecular microarchitecture. Calibration and quality control were performed weekly using five density standards, and spatial resolution was verified monthly using a tungsten wire rod. Beam hardening correction was based on the calibration records.

### Histomorphometry

Vertebrae (L1–L3) were fixed in 10% Millonig’s formalin for 24 h and gradually transferred to 100% ethanol. The undecalcified bone samples were then embedded in methyl methacrylate and 5 µm thick sections were prepared. Histomorphometric analysis of osteoclasts and osteoblasts was performed on TRAP and toluidine blue-stained longitudinal sections using the OsteoMeasure Analysis System (OsteoMetrics Inc., Decatur, GA). All the measurements were restricted to the secondary spongiosa. Terminology used was recommended by the Histomorphometry Nomenclature Committee of the American Society for Bone and Mineral Research^30^.

### Statistics

One-way ANOVA or Student’s *t* test was used to detect statistically significant treatment effects, after determining that the data were normally distributed and exhibited equivalent variances. In some cases, log transformation was used to obtain normally distributed data. All *t* tests were two sided. Bonferroni or Holm-Sidak corrections were used for multiple comparisons. *P* values less than 0.05 were considered significant. Sample sizes for animal experiments were selected based on previous experiments that had sufficient power to detect the expected effect size^3^. No animals or samples were excluded from the analyses. Femurs or vertebra that were physically damaged during harvest were not analyzed by microCT or histology. All individuals involved in sample analysis were blinded to the group identity of the sample.

### Data availability

The data sets generated during and/or analyzed during the current study are available from the corresponding author on reasonable request.

## Electronic supplementary material

Supplementary Information
