# Supplementary material for: Soluble RANKL contributes to osteoclast formation in adult mice but not ovariectomy-induced bone loss
Source: Nat Commun. 2018 Jul 25;9:2909. doi: 10.1038/s41467-018-05244-y (PMC6060116; doi:10.1038/s41467-018-05244-y)
Supplement: Supplementary file 1 — Supplementary Information [file 41467_2018_5244_MOESM1_ESM.pdf]

Soluble RANKL contributes to osteoclast formation in adult mice but not ovariectomy-induced bone loss

Xiong et al

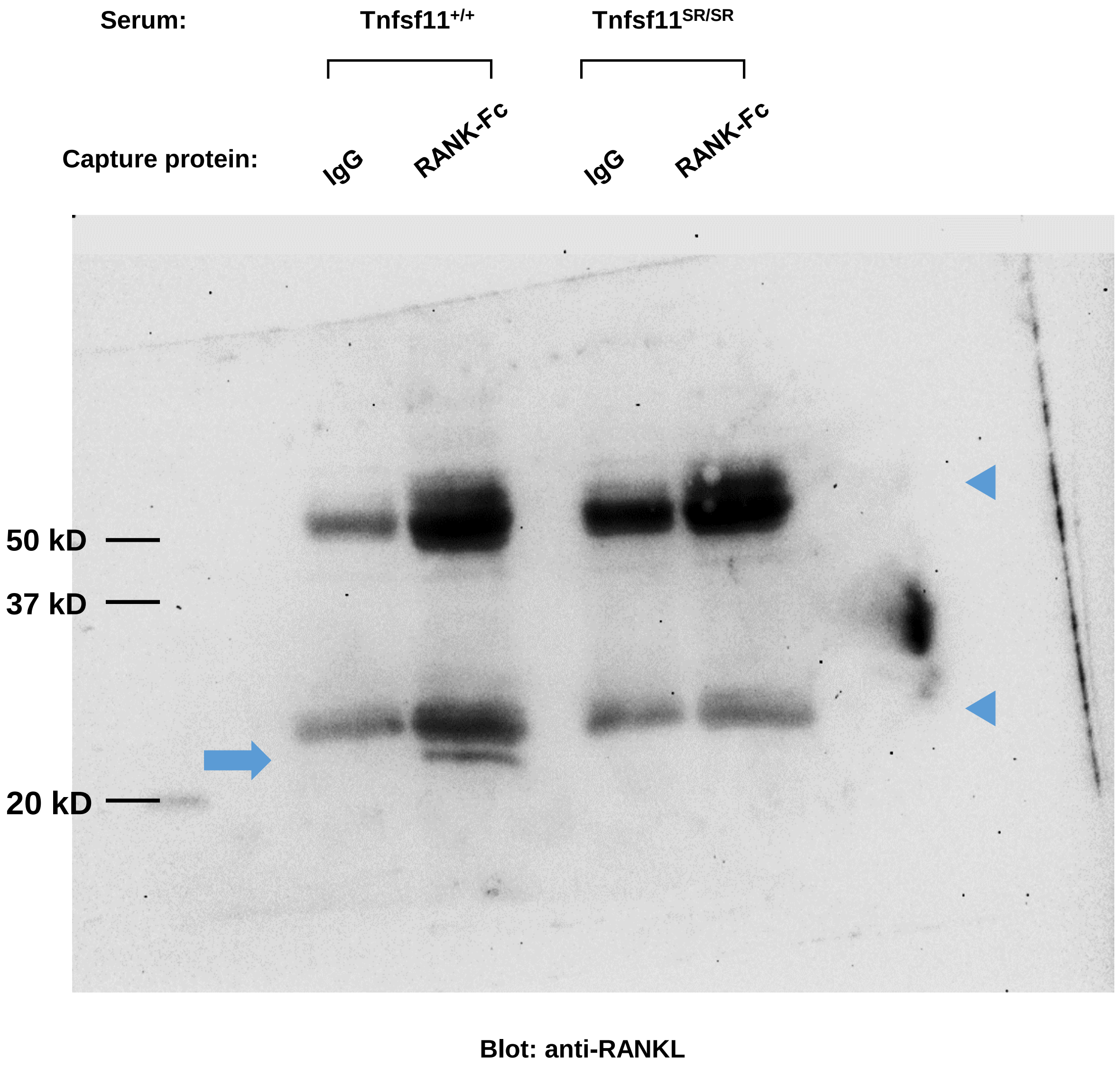

Supplementary Figure 1. Tnfsf11$^{SR/SR}$ mice lack sRANKL as measured by affinity isolation. Analysis of sRANKL from the serum of Tnfsf11$^{+/+}$ and Tnfsf11$^{SR/SR}$ mice by affinity isolation and immunoblot. The arrow indicates the location of sRANKL and the arrowheads indicate the location of IgG heavy and light chains.

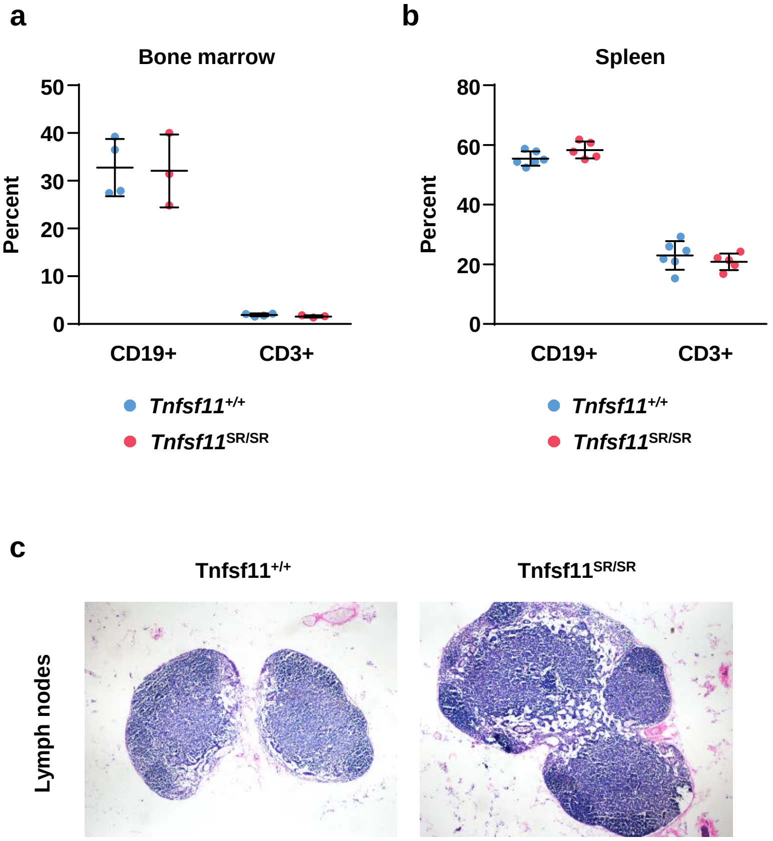

Supplementary Figure 2. Tnfsf11$^{SR/SR}$ mice display normal lymphocyte and lymph node development. (**a**) Quantification of B lymphocytes (CD19+) and T lymphocytes (CD3+) from bone marrow of Tnfsf11$^{+/+}$ (*n* = 4) and Tnfsf11$^{SR/SR}$ (*n* = 3) mice (values are mean ± s.d.). (**b**) Quantification of B lymphocytes (CD19+) and T lymphocytes (CD3+) from the spleen of Tnfsf11$^{+/+}$ (*n* = 6) and Tnfsf11$^{SR/SR}$ (*n* = 5) mice (values are mean ± s.d.). (c) Images of histological sections of inguinal lymph nodes from mice of the indicated genotypes.

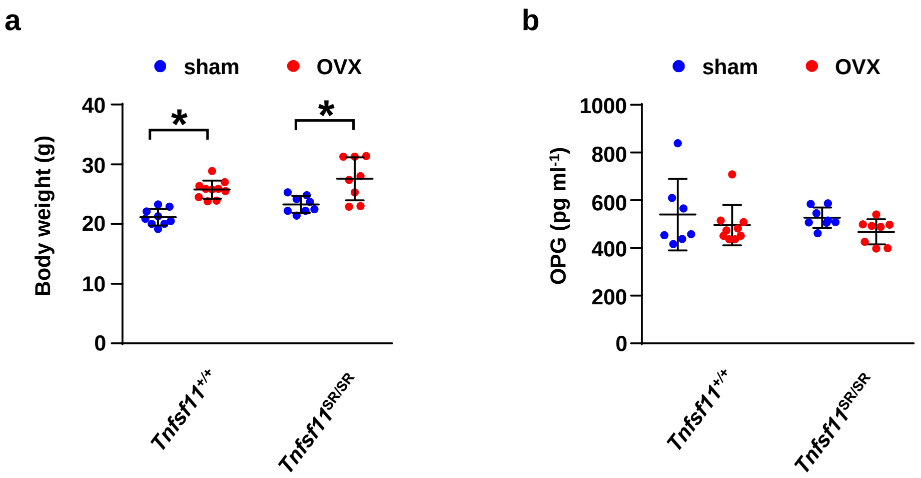

Supplementary Figure 3. Body weight increases, but circulating OPG levels are unchanged, by ovariectomy in Tnfsf11$^{+/+}$ and Tnfsf11$^{SR/SR}$ mice. Body weight (**a**), and OPG protein levels in the serum (**b**), of sham-operated (sham) and ovariectomized (ovx) Tnfsf11$^{+/+}$ (*n* = 7 and 9 for sham and ovx, respectively) and Tnfsf11$^{SR/SR}$ (*n* = 8 and 8 for sham and ovx, respectively). *P<0.05 using Student's t-test.

Flow cytometry gating strategy

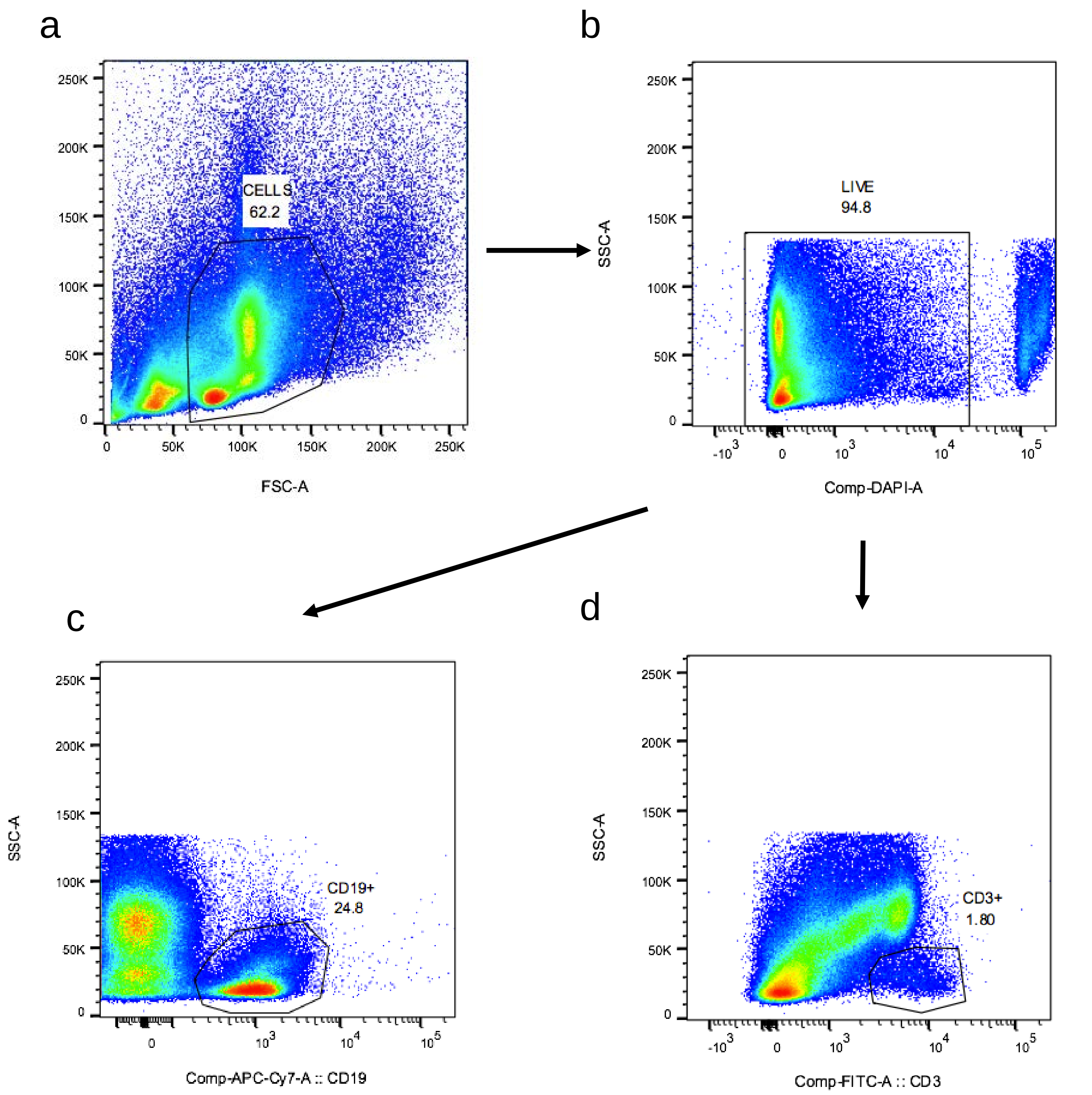

Supplementary Figure 4. Representative gating strategy of a bone marrow sample. Cells were gated on a forward scatter (FSC)/side scatter (SSC) plot (**a**), excluding cellular debris, and then live cells were selected by gating on the cells that excluded the viability dye (DAPI-negative) (**b**). B cells were recognized by gating on side scatter (SSC)/CD19-APC (**c**). T cells were recognized by gating on side scatter (SSC)/CD3-FITC (**d**). Percentages of sub-populations are shown for each graph.

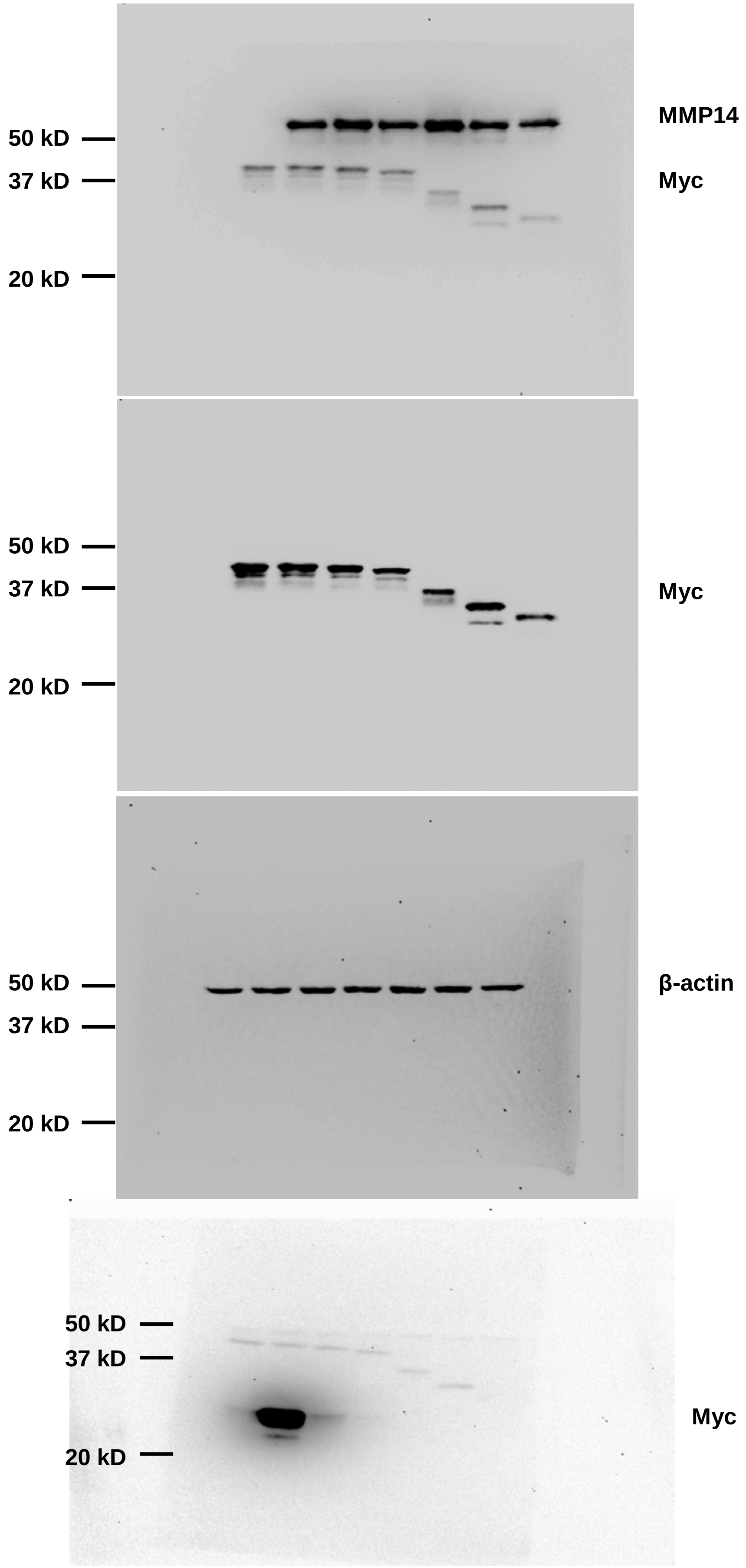

Supplementary Figure 5. Uncropped western bots used in Fig. 1b.

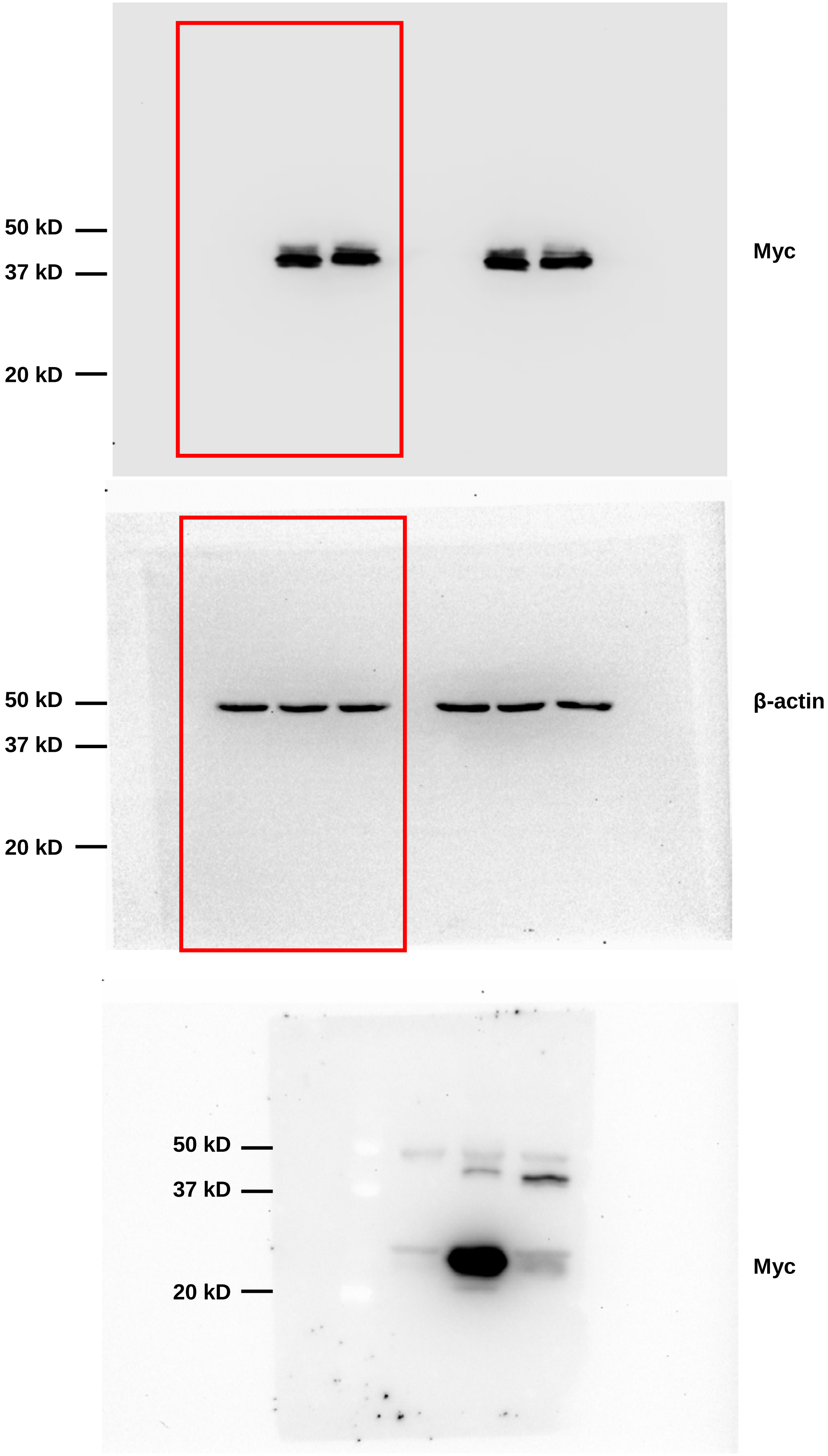

Supplementary Figure 6. Uncropped western bots used in Fig. 1c. Red box indicates the bands showed in Fig. 1c.

Supplementary Table 1

Primer Sequences			
No.	Name	Sequence	Product Size (bp)
1	ex3OT1-for<br>ex3OT1-rev	cagggatccCCCCTTTCTCCGTAAGAAGC<br>caggtcgacTGCATCATCATGGAACATCA	360
2	ex3OT2-for<br>ex3OT2-rev	cagggatccTGTCCCCACCATCCTTTAAT<br>caggtcgacTGGTAAGCCAGAAGGCTCAT	325
3	ex3OT3-for<br>ex3OT3-rev	CATGCTTCAGGGATCCTCAT<br>caggtcgacGGTTTGGTCCTCACTGGTCA	354
4	ex3OT4-for<br>ex3OT4-rev	cagggatccGGACATTTGGGACTGGGTAA<br>caggtcgacTTTTGAAGCCATTCCTGACC	320
5	ex3OT5-for<br>ex3OT5-rev	cagggatccCCACAACCCCTTACAGGATG<br>caggtcgacAAGGAAAAGTAGCCCCAGGT	319
6	ex3OT6-for<br>ex3OT6-rev	cagggatccGCCACTGGCACTTATCAAAA<br>caggtcgacGCTCTGGAAAAATCTTGCTCAT	444
7	ex4OT1-for<br>ex4OT1-rev	cagggatccCTGACACAGAAGCGGCACTA<br>caggtcgacGGACTGCAGCTCTCAGCTCT	318
8	ex4OT2-for<br>ex4OT2-rev	cagggatccTTTTTCTTGGCCATTTCACA<br>caggtcgacGCAGATAATTAAGCCAGGCAAC	329
9	ex4OT3-for<br>ex4OT3-rev	cagggatccTTTGCCATAGACTGTTTTCTTG<br>caggtcgacCATCCCTCAGGAGCAAGTTC	323
10	ex4OT4-for<br>ex4OT4-rev	cagggatccTTTTCTTTGACCCAGTATTACCA<br>caggtcgacTCTGAGTTCCCTGCACAATG	305
11	ex4OT5-for<br>ex4OT5-rev	cagggatccTTCCAAAAGGAGCCTTAAGAAG<br>caggtcgacCAGGCCAAAATGTCAAAAGA	402
12	ex4OT6-for<br>ex4OT6-rev	cagggatccGGGACATTTCTGAGGACACAA<br>caggtcgacAGCAGCACAGGGCATAGTG	349
13	ex4OT1mu-for<br>ex4OT1mu-rev	CTGACACAGAAGCGGCACTA<br>CCTTTCATCCCATTTATGAGC	160

Supplementary Table 2

Summary of Off-target Cleavage				
Sites	Sequence	Indel Frequency (Mutant/Total)	Locus	Overlapping Genes
Exon 3 sgRNA	TCAGGAACTGCAACACATTGTGG	0/5	chr14:78284445-78284470	No
Ex3OT1	GCAGTAAATTCAACACATTGTGG	0/5	chr8:96842918-96842943	No
Ex3OT2	TGAGGAGCTGCAACACATTCTGG	0/5	chrX:59562222-59562247	No
Ex3OT3	TAAGGAACTACAACACATAGTGG	0/5	chr14:13074161-13074186	No
Ex3OT4	TTACCACCTGCAACACATTGAAG	0/5	chr7:26571291-26571316	Yes
Ex3OT5	TCAAGAACAGAAACACATTGGGG	0/5	chrX:136094828-136094853	No
Ex3OT6	TAAAGCATTGCAACACATTGCAG	0/5	chr13:82396661-82396686	No
Exon 4 sgRNA	TATGATGGAAGGCTCATGGTTGG	0/5	chr14:78284312-78284337	No
Ex4OT1	TAAGAAGGAAGGCTCATGGTTGG	2/5	chr12:71247138-71247163	No
Ex4OT2	TCTGATGAAAGGCTCATGGAAAG	0/5	chr5:98995982-98996007	No
Ex4OT3	TCTGGTGGAAGCCTCATGGTAGG	0/5	chr14:35355852-35355877	No
Ex4OT4	TATGCTGGACAGCTCATGGTAGG	0/5	chr9:95577402-95577427	No
Ex4OT5	CATGTTGGAAGGCTCATGATCAG	0/5	chr14:102856819-102856844	No
Ex4OT6	TGTGAAGGAAAGCTCATGGTTAG	0/5	chr1:93292054-93292079	No

Supplementary Table 3

Sequences of cloned off-target PCR products		
	Exon 3	Exon 4
OT 1	GAGACTGCAGTAAATTCAACACATTGTGGTTTGGA<br>GAGACTGCAGTAAATTCAACACATTGTGGTTTGGA<br>GAGACTGCAGTAAATTCAACACATTGTGGTTTGGA<br>GAGACTGCAGTAAATTCAACACATTGTGGTTTGGA<br>GAGACTGCAGTAAATTCAACACATTGTGGTTTGGA<br>GAGACTGCAGTAAATTCAACACATTGTGGTTTGGA<br>chr8:96842918-96842943 Overlapping Genes: None	ACCAAGTAAGAAGGAAGGCTCATGGTTGGATGATG<br>ACCAAGTAAGAAGGAAGGCTCATGGTTGGATGATG<br>ACCAAGTAAGAAGGAAGGCTCATA-----A--ATG<br>ACCAAGTAAGAAGGAAGGCTCATGGTTGGATGATG<br>ACCAAGTAAGAAGGAAGGCTCATGGTTGGATGATG<br>ACCAAGTAAGAAGGAAGGCTCATA-----A--ATG<br>chr12:71247130-71247172 Overlapping Genes: None
OT 2	TATATTTGAGGAGCTGCAACACATTCTGGTTGTTC<br>TATATTTGAGGAGCTGCAACACATTCTGGTTGTTC<br>TATATTTGAGGAGCTGCAACACATTCTGGTTGTTC<br>TATATTTGAGGAGCTGCAACACATTCTGGTTGTTC<br>TATATTTGAGGAGCTGCAACACATTCTGGTTGTTC<br>TATATTTGAGGAGCTGCAACACATTCTGGTTGTTC<br>chrX:59562222-59562247 Overlapping Genes: None	AGTGACTCTGATGAAAGGCTCATGGAAAGAAGAGG<br>AGTGACTCTGATGAAAGGCTCATGGAAAGAAGAGG<br>AGTGACTCTGATGAAAGGCTCATGGAAAGAAGAGG<br>AGTGACTCTGATGAAAGGCTCATGGAAAGAAGAGG<br>AGTGACTCTGATGAAAGGCTCATGGAAAGAAGAGG<br>AGTGACTCTGATGAAAGGCTCATGGAAAGAAGAGG<br>chr5:98995982-98996007 Overlapping Genes: None
OT 3	TTGTGATAAGGAACTACAACACATAGTGGATCACG<br>TTGTGATAAGGAACTACAACACATAGTGGATCACG<br>TTGTGATAAGGAACTACAACACATAGTGGATCGCG<br>TTGTGATAAGGAACTACAACACATAGTGGATCACG<br>TTGTGATAAGGAACTACAACACATAGCGGATCACG<br>TTGTGATAAGGAACTACAACACATAGTGGATCACG<br>chr14:13074161-13074186 Overlapping Genes: None	GTAGTTTCTGGTGGAAGCCTCATGGTAGGTAGCAT<br>GTAGTTTCTGGTGGAAGCCTCATGGTAGGTAGCAT<br>GTAGTTTCTGGTGGAAGCCTCATGGTAGGTAGCAT<br>GTAGTTTCTGGTGGAAGCCTCATGGTAGGTAGCAT<br>GTAGTTTCTGGTGGAAGCCTCATGGTAGGTAGCAT<br>GTAGTTTCTGGTGGAAGCCTCATGGTAGGTAGCAT<br>chr14:35355852-35355877 Overlapping Genes: None
OT 4	TGGTTCTTACCACCTGCAACACATTGAAGAGCTTA<br>TGGTTCTTACCACCTGCAACACATTGAAGAGCTTA<br>TGGTTCTTACCACCTGCAACACATTGAAGAGCTTA<br>TGGTTCTTACCACCTGCAACACATTGAAGAGCTTA<br>TGGTTCTTACCACCTGCAACACATTGAAGAGCTTA<br>TGGTTCTTACCACCTGCAACACATTGAAGAGCTTA<br>chr7:26571291-26571316 Overlapping Genes: Yes	TGCCTATATGCTGGACAGCTCATGGTAGGTGTTCT<br>TGCCTATATGCTGGACAGCTCATGGTAGGTGTTCT<br>TGCCTATATGCTGGACAGCTCATGGTAGGTGTTCT<br>TGCCTATATGCTGGACAGCTCATGGTAGGTGTTCT<br>TGCCTATATGCTGGACAGCTCATGGTAGGTGTTCT<br>TGCCTATATGCTGGACAGCTCATGGTAGGTGTTCT<br>chr9:95577402-95577427 Overlapping Genes: None
OT 5	ATTTCGTCAAGAACAGAAACACATTGGGGAACATA<br>ATTTCGTCAAGAACAGAAACACATTGGGGAACATA<br>ATTTCGTCAAGAACAGAAACACATTGGGGAACATA<br>ATTTCGTCAAGAACAGAAACACATTGGGGAACATA<br>ATTTCGTCAAGAACAGAAACACATTGGGGAACATA<br>ATTTCGTCAAGAACAGAAACACATTGGGGAACATA<br>chrX:136094828-136094853 Overlapping Genes: None	ACTCGACATGTTGGAAGGCTCATGATCAGCTCCTC<br>ACTCGACATGTTGGAAGGCTCATGATCAGCTCCTC<br>ACTCGACATGTTGGAAGGCTCATGATCAGCTCCTC<br>ACTCGACATGTTGGAAGGCTCATGATCAGCTCCTC<br>ACTCGACATGTTGGAAGGCTCATGATCAGCTCCTC<br>ACTCGACATGTTGGAAGGCTCATGATCAGCTCCTC<br>chr14:102856819-102856844 Overlapping Genes: None
OT 6	TGATAATAAAGCATTGCAACACATTGCAGCTCTAT<br>TGATAATAAAGCATTGCAACACATTGCAGCTCTAT<br>TGATAATAAAGCATTGCAACACATTGCAGCTCTAT<br>TGATAATAAAGCATTGCAACACATTGCAGCTCTAT<br>TGATAATAAAGCATTGCAACACATTGCAGCTCTAT<br>TGATAATAAAGCATTGCAACACATTGCAGCTCTAT<br>chr13:82396661-82396686 Overlapping Genes: None	ACACAATGTGAAGGAAAGCTCATGGTTAGCGGTTT<br>ACACAATGTGAAGGAAAGCTCATGGTTAGCGGTTT<br>ACACAATGTGAAGGAAAGCTCATGGTTAGCGGTTT<br>ACACAATGTGAAGGAAAGCTCATGGTTAGCGGTTT<br>ACACAATGTGAAGGAAAGCTCATGGTTAGCGGTTT<br>ACACAATGTGAAGGAAAGCTCATGGTTAGCGGTTT<br>chr1:93292054-93292079 Overlapping Genes: None

*Dashes (-) represent deleted bases
